# Inflation Pressure in Side Branch during Modified Jailed Balloon Technique Does Not Affect Side Branch Outcomes

**DOI:** 10.1155/2021/8839897

**Published:** 2021-02-18

**Authors:** Tetsuya Nomura, Naotoshi Wada, Issei Ota, Satoshi Tasaka, Kenshi Ono, Yu Sakaue

**Affiliations:** Department of Cardiovascular Medicine, Kyoto Chubu Medical Center, Nantan, Japan

## Abstract

**Objectives:**

This study aimed to investigate the optimal jailed balloon inflation in the side branch during the modified jailed balloon technique for bifurcated lesions.

**Background:**

The modified jailed balloon technique is one of the effective techniques to minimize the emergence of side branch (SB) compromise by preventing plaque or carina shifting during a single stent strategy in the main vessel with provisional SB treatment. However, there are no detailed studies on the method of optimal jailed balloon inflation.

**Methods:**

We analyzed 51 consecutive patients who underwent percutaneous coronary intervention (PCI) for bifurcated lesions with a modified jailed balloon technique between September 2018 and December 2020. These 51 patients were divided into two groups according to the magnitude of inflation pressure of the jailed balloon: a higher pressure (HP) group and lower pressure (LP) group.

**Results:**

No significant differences in procedural outcomes were observed between the two groups. The findings of SB compromise were relatively common with our procedure (30.0% in the HP group; 33.3% in the LP group). The patterns of SB compromise such as dissection or stenosis increase were observed at similar frequencies between them. In particular, SB dissection was noted in the SB lesion with some plaque burden, irrespective of the magnitude of the jailed balloon inflation pressure. Univariate analysis showed that calcification in the main vessel and SB lesion length was significantly associated with SB compromise. Finally, all PCI procedures were successfully completed without any provisional stent deployment in SB.

**Conclusions:**

We speculate that lesion characteristics rather than the PCI procedural factors may be critical determinants to cause SB compromise.

## 1. Introduction

Even in this modern era of interventional cardiology, an optimal percutaneous coronary intervention (PCI) strategy for bifurcated lesions, which accounts for 15–20% of all PCI, remains controversial [1, 2]. At present, there is a consensus that a single stent strategy only for the main vessel (MV) with provisional side branch (SB) treatment is a preferable technique for the majority of bifurcated lesions [3]. During a provisional approach, stenting in the MV crossing over the SB often aggravates SB ostial stenosis, leading to SB compromise, which results in periprocedural myocardial infarction, one of the most serious procedure-related complications [4]. Previously, the main mechanism of this complication was considered to be plaque shift from MV toSB [5]. However, a pathological study showed that atherosclerotic plaques are mostly generated on the lateral wall, whereas the bifurcated carina tends to be spared, which indicates that the contribution of plaque shift has been overestimated [6]. Instead, the carina itself can be shifted to SB, which is now considered to be the major cause of anatomical SB compromise [7].

Therefore, strategies are needed to prevent SB compromise during a provisional stenting procedure. Preparing the second guidewire in the SB during MV stenting is usually performed in this clinical setting but cannot completely prevent SB compromise (jailed wire technique) [8]. In 2010, Burzotta et al. reported an epoch-making novel technique “jailed balloon protection” to avoid SB occlusion during provisional stenting of bifurcated lesions [9]. Although the jailed balloon was not initially inflated in their procedure, SB occlusion was reduced due to the effect of spatial occupation of the SB ostium. However, this procedure also cannot completely prevent SB compromise [10]. Since then, various modified techniques derived from the original jailed balloon technique have been reported. The balloon-stent kissing technique (BSKT) involves inflating a balloon with a suitable pressure in SB during MV stent deployment [11, 12]. The jailed semi-inflated balloon technique (JSBT) is a procedure in which, on the basis of BSKT, the proximal optimization technique (POT) is performed for optimization for MV stent apposition [13, 14]. Compared with the jailed balloon technique, the jailed Corsair technique is more suitable for protecting a small SB, and the risk of causing SB ostial dissection is reduced [15].

Saito et al. demonstrated the modified jailed balloon technique in 2018, which was safe and effective for the preservation of SB patency during bifurcation stenting [16]. Their method is characterized in three ways. First, the proximal end of the jailed balloon should be positioned at the SB ostium, attaching to the stent being deployed in the MV. Second, the diameter of the jailed balloon is determined to be half of the stent diameter in the MV. Third, both the jailed balloon and MV stent are dilated simultaneously to around 12 atmospheres. They reported that this inflation procedure prevents occlusion of the SB ostium by preventing plaque or carina shifting during MV stent implantation. However, as many as 12.2% of their cases needed T and protrusion (TAP) stenting after the modified jailed balloon technique. The reason why TAP stenting was required is not discussed in the paper, but jailed balloon inflation to around 12 atmospheres in the diseased SB might have affected the outcomes. To date, there have been no detailed studies on the method of optimal jailed balloon inflation in SB during the modified jailed balloon technique. Therefore, we aim to discuss it in the present study.

## 2. Materials and Methods

This is a retrospective observational study conducted at Kyoto Chubu Medical Center, Nantan, Japan. The study protocol was approved by the institutional ethics committee, and all patients who underwent PCI provided written informed consent for the PCI procedure and the management of their data.

Between September 2018 and December 2020, patients who received PCI for coronary bifurcated lesions with organic atheromatous coronary stenosis or occlusion, which was verified as the cause of myocardial ischemia or acute coronary syndrome, were enrolled in this study. We targeted bifurcations with a vessel size of ≥2.5 mm in MV and ≥2.0 mm in SB on visual estimation on coronary angiography (CAG). Each coronary bifurcated lesion was classified according to the Medina classification [17]. PCI procedures including how to protect SB and devise a stenting strategy were left to the operator's discretion. Among them, we selected cases in which we conducted a modified jailed balloon technique. We did not define the precise rule regarding case selection that needed a modified jailed balloon technique for SB protection in the provisional single stent strategy. However, all operators were judged to apply this technique considering the risky situations of SB compromise, which had been reported previously [18, 19]. Those are situations such as narrower bifurcation angle, stenosed SB ostium, or plaque distribution in the MV wall at the opposite side of the SB.

Baseline characteristics of study patients including age, sex, hypertension (HT), diabetes mellitus (DM), dyslipidemia, current smoking status, use of hemodialysis (HD), paroxysmal or chronic atrial fibrillation, comorbidity of cerebral vascular disease (CVD) or peripheral arterial disease (PAD), and medications were recorded. HT was defined as systemic blood pressure ≥140/90 mmHg or the presence of antihypertensive treatment; DM was defined as fasting blood sugar ≥126 mg/dL or the presence of specific treatment; dyslipidemia was defined as total cholesterol ≥220 mg/dL or the presence of cholesterol-lowering agents.

CAG images were displayed and recorded in a GoodView PRO workstation (GOODMAN Co., Ltd., Aichi, Japan), and we compared lesion characteristics and procedural outcomes in the study patients by analyzing the records and angiography films of the PCI.

### 2.1. Procedure

Study patients received 100 mg of aspirin and 75 mg of clopidogrel or 3.75 mg of prasugrel prior to coronary intervention. Just after sheath insertion, an intra-arterial bolus of 8,000 units of unfractionated heparin was administered, and the activated clotting time was controlled to over 250 seconds during the procedure. We adopted the concepts of the modified jailed balloon technique described in Saito et al.'s paper [16]. In summary, after two guidewires are advanced in both MV and SB, diseased vessels are predilated as necessary. The MV stent is initially delivered to the deployment position crossing over SB, and then the jailed balloon is introduced into SB. The position of the jailed balloon is carefully adjusted as its proximal end is attached to the MV stent, and the jailed balloon diameter is fundamentally determined according to the SB lumen size by visual estimation in CAG. When deploying a stent, we simultaneously inflate both the stent in MV and the jailed balloon in SB two or three times. In this step, the magnitude of the inflation pressure of the jailed balloon catheter was left to the operator's discretion. In the higher pressure (HP) group, we usually inflate both the stent and jailed balloon at a nominal pressure of the stent balloon. On the other hand, in the lower pressure (LP) group, the MV stent is deployed at a nominal inflation pressure, and at the same time, the jailed balloon is usually inflated at a nominal pressure of the semicompliant jailed balloon. After removing both balloons, we pass the third guidewire to SB as distally as possible using a dual lumen microcatheter in all cases. Postballoon inflation in the MV stent using an adequate size is mandatory according to the degree of stent expansion. Additional balloon inflation in SB, kissing balloon inflation, or POT is performed based on the findings of angiography and/or intravascular imaging modalities. If necessary, additional stent deployment in SB is considered.

### 2.2. Quantitative Coronary Angiography Analysis

Quantitative coronary angiography (QCA) analyses were performed for both MV and SB of the targeted bifurcation from both the initial and final angiography, respectively. CAG images obtained by Trinias C12 (Shimadzu, Tokyo, Japan) were submitted to QAngio XA (Medis, Leiden, The Netherlands) software for QCA analysis. According to the algorithm in the dedicated software, the reference vessel diameter, minimal lumen diameter, percent diameter stenosis, and lesion length were measured in MV and SB on QCA. The outer diameter of the contrast-filled catheter was used for calibration, and QCA analysis was performed from the single-best-available projection with the least foreshortening and the clearest bifurcation.

### 2.3. Definitions

SB compromise was defined as follows. If SB ostial stenosis was <50.0% before MV stenting, then it was defined as SB stenosis increase to >50.0%, SB Thrombolysis In Myocardial Infarction (TIMI) flow grade <grade 3, or SB ostial dissection ≥NHLBI type B. If SB ostial stenosis was ≥50.0% before MV stenting, then it was defined as stenosis increase, SB TIMI flow <grade 3, or SB ostial dissection ≥NHLBI type B [20]. The NHLBI classification system for coronary artery dissection is defined as an intraluminal filling defect, extravasation of contrast material, and linear density of staining [21].

### 2.4. Statistical Analysis

Descriptive statistics are presented as *n* (%) for categorical variables. Baseline characteristics were compared using a chi-squared or Fischer's exact test for categorical variables and an unpaired Student's *t*-test for continuous variables. For nonnormally distributed continuous variables, the Mann–Whitney *U* test was used. The factors associated with SB compromise in the univariate analysis were examined by multivariate logistic regression analysis. The odds ratio (OR) and 95% confidence interval (CI) assessing the risk of SB compromise were recorded. Cutoff values about parameters related to lesion characteristics were assessed by receiver operating characteristics (ROC) curve analysis. A *P* value of <0.05 was considered to represent a significant difference. All statistical models were constructed using R Programming Language.

## 3. Results

Between September 2018 and December 2020, we encountered a total of 713 consecutive PCI cases. Among them, 200 (28.1%) cases included bifurcated lesions as targets for intervention. Of these, we adopted a modified jailed balloon technique to prevent SB compromise for 51 bifurcated lesions in 51 cases (7.2% of total PCI cases) in the procedure of a single stent provisional T stenting strategy. Each of the HP and LP groups included 30 and 21 cases, respectively.

Baseline clinical characteristics are shown in Table 1. No significant difference was observed in the baseline clinical characteristics between the two groups. The angiographic lesion characteristics were also similar in each group (Table 2). Most of the targeted lesions were located in the bifurcation of the left anterior descending artery (LAD) and diagonal branch (80.0% of cases in the HP group, 90.5% of the LP group). The Medina classification was as follows: (1, 1, 1) 18 cases in the HP group and 14 cases in the LP group, (1, 1, 0) 1 case in each group, (1, 0, 1) 4 cases in the HP group and 2 cases in the LP group, (0, 1, 1) 6 cases in the HP group and 4 cases in the LP group, and (0, 0, 1) 1 case in the HP group. The rate of true bifurcation was 93.3% in the HP group and 95.2% in the LP group. Details of the PCI procedures were compared, and there was no significant difference between the two groups except for the diameter size of the guide catheter and jailed balloon inflation pressure in SB (Table 2). In the HP group, the jailed balloon inflation pressure was 10.8 ± 1.9 atm, which was the same as the MV stent inflation pressure. On the other hand, it was 5.4 ± 2.4 atm in the LP group, which was significantly lower than that in the HP group (*P* < 0.05) (Table 2).

Procedural outcomes are shown in Table 3. Although we successfully performed PCI procedures for bifurcated lesions in all 51 cases without any flow disturbance in SB, the findings of SB compromise were relatively frequent with our procedure (30.0% in the HP group; 33.3% in the LP group). The patterns of SB compromise such as dissection or stenosis increase were observed at similar frequencies in the two groups. However, even in this situation, we could pass a guidewire to the jailed SB through the stent strut except for 1 case in each group. Extrastent dilatation to compress the SB dissection to allow the guidewire to recross to SB was needed in 1 case in the HP group. A transiently reduced SB TIMI flow grade (<TIMI 3) due to SB compromise was observed in 1 case in the HP group and 2 cases in the LP group. However, the flow disturbance could be overcome by balloon inflation without provisional stent deployment. No jailed balloon or guidewire entrapment by the MV stent occurred in any case. As a result, there were no significant differences in procedural outcomes between the two groups in our study. No in-hospital major adverse cardiac event (MACE) including procedural myocardial infarction occurred during the hospital stay.

Quantitative coronary angiographic (QCA) analysis is shown in Table 4. No significant differences in the parameters between the two groups were observed in the initial or final angiography. Predictive factors for SB compromise were assessed and shown in Table 5. Cutoff values about parameters related to SB lesion characteristics were calculated in ROC analysis as follows: SB reference vessel diameter (RVD); 1.79-mm (sensitivity, 81.2%; specificity, 54.3%), SB minimal lumen diameter (MLD); 0.54-mm (sensitivity, 62.5%; specificity, 62.9%), SB lesion length (LL); 8.65-mm (sensitivity, 56.2%; specificity, 77.1%). Univariate analysis of several parameters related to PCI procedure or lesion characteristics showed that MV calcification which was fluoroscopically recognized at the bifurcation (OR 9.65 [95% CI, 1.21–449.44], *P*=0.019) and SB lesion length ≥8.65-mm (OR 4.20 [95% CI, 1.03–18.48], *P*=0.027) was significantly associated with SB compromise. However, their statistical significance was cancelled in the multivariate analysis (MV calcification: OR 8.74 [95% CI, 0.99–76.90], *P*=0.051, SB lesion length ≥8.65-mm (OR 3.77 [95% CI, 0.99–14.40], *P*=0.052).

**Figure 1:**
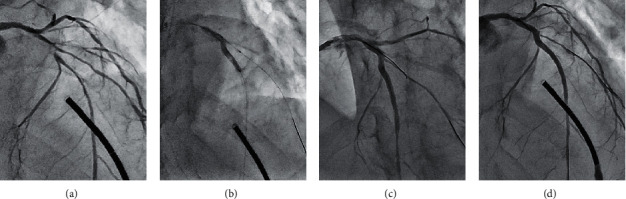
A case of stenosis increase in the SB ostium after the modified jailed balloon technique. (a) A control angiographic image of the targeted bifurcated lesion in the mid-LAD artery. (b) DES deployment simultaneously with 1.5 mm jailed balloon inflation in SB. (c) After the modified jailed balloon technique, arterial dissection developed in the SB ostium. (d) Final angiography with stenosis increase in the SB ostium.

**Figure 2:**
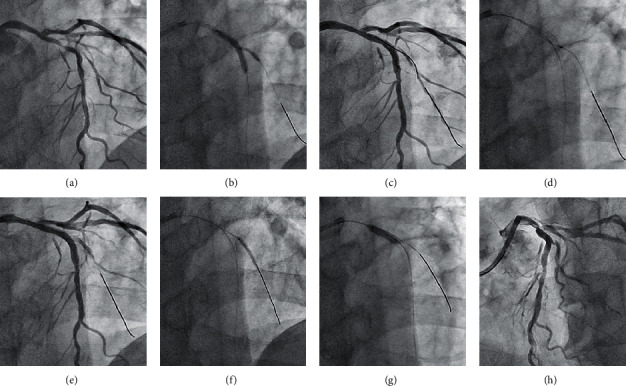
A case of severe dissection in the SB ostium after the modified jailed balloon technique. (a) A control angiographic image of the targeted bifurcated lesion in the mid-LAD artery. (b) DES deployment simultaneously with 2.0 mm jailed balloon inflation in SB. (c) Recrossed guidewire advancement in the dissected lumen of SB. (d) Extrastent balloon dilatation with a 1.5 mm balloon to compress the dissected lumen. (e) Successful guidewire passage in SB with limited blood flow. (f) Vessel fenestration at the lesion of hematoma with a cutting balloon. (g) POT with a 3.0 mm noncompliant balloon catheter. (h) Final angiography with favorable blood flow both in MV and SB.

We show two typical cases with SB compromise after the modified jailed balloon technique.

  (Case 1) PCI was performed for the bifurcated lesion (Medina 1, 1, 1) in the mid-LAD artery of a 70-year-old male (Figure 1(a)). After 2.5 mm balloon inflation in the LAD artery, a SYNERGY 3.0/28 mm (Boston Scientific, MA, USA) drug eluting stent (DES) was deployed at a 12 atm inflation pressure simultaneously with 1.5 mm jailed balloon inflation in SB at a 6 atm pressure (Figure 1(b)). Arterial dissection occurred at the ostium of the diagonal branch and a guidewire to recross was initially easy to advance in the dissected lumen (Figure 1(c)). Finally, a Fielder FC (ASAHI INTECC Co., Ltd., Aichi, Japan) guidewire could be successfully passed to SB and kissing balloon inflation was performed. Although TIMI3 flow in SB was achieved, increased stenosis was noted at the SB ostium (% diameter stenosis increased from 66.0 to 81.8%) (Figure 1(d)).
  (Case 2) A 62-year-old male with stable angina pectoris underwent PCI for Medina (1,0,1) bifurcation in the mid-LAD artery (Figure 2(a)). After vessel preparation with 2.5 mm balloon inflation in MV, we implanted an Orsiro 3.0/30-mm (Japan Lifeline Co., Ltd., Tokyo, Japan) DES with 2.0 mm jailed balloon inflation in SB simultaneously at an 8 atm pressure (Figure 2(b)). A dissection also occurred at the ostium of the diagonal branch in this case, and we could advance the third guidewire only in the dissected lumen (Figure 2(c)). Thereafter, stasis of the blood flow due to enlargement of the dissected lumen was observed, and we performed extrastent balloon dilatation with a 1.5 mm balloon to compress the dissected lumen (Figure 2(d)). Then, we could advance a Wizard 78 (Japan Lifeline Co., Ltd., Tokyo, Japan) guidewire exactly in SB, although stasis of the blood flow continued due to a vessel hematoma in the middiagonal artery (Figure 2(e)). We performed vessel fenestration at the lesion of the hematoma with 2.0 mm Wolverine cutting balloon (Boston Scientific, MA, USA) inflation (Figure 2(f)). After POT with 3.0 mm noncompliant balloon inflation (Figure 2(g)), favorable blood flow both in MV and SB was achieved based on the final angiography (Figure 2(h)).

## 4. Discussion

The present study describes 51 sequential cases in which we performed the modified jailed balloon technique in PCI for bifurcated lesions. The procedure of the modified jailed balloon technique in our hospital is fundamentally conducted according to Saito's original one [16]. This inflation procedure is clearly different from the conventional modified jailed balloon technique in that the proximal end of the jailed balloon is positioned at the SB ostium, which can lead to minimal interference with the MV stent. Also, it can minimize the risk of occlusion in the SB ostium through preventing plaque or carina shifting during MV stent implantation due to the effect of spatial occupation of the SB ostium.

After the modified jailed balloon technique, the presence of stent malpositioning proximal to the bifurcation is usually observed due to the mass effect of the jailed balloon shaft outside the stent, so additional balloon inflation at high pressure in the MV stent is essential for optimal stent expansion. However, the procedure is associated with the risk of worsening SB compromise, and preceding that procedure, recrossing a guidewire to SB through the MV stent strut is desirable for easy access to SB in case of SB compromise. On the other hand, jailed balloon inflation in SB is associated with the risk of causing arterial dissection, especially in cases of a diseased SB. In the presence of a dissected SB, we have to take care when performing guidewire recross into SB to avoid enlarging the dissected lumen. Saito's paper showed that as many as 12.2% of their cases required TAP stenting after the modified jailed balloon technique. Their maneuver of jailed balloon inflation to around 12 atmospheres in the diseased SB might have caused severe SB dissection and necessitated additional stent deployment in SB. Therefore, we noted how we could minimize the risk of arterial dissection of SB and prevent plaque or carina shifting during MV stent implantation.

We considered that three factors associated with jailed balloon inflation might affect SB compromise: diameter, length, and inflation pressure of the jailed balloon. In this study, we essentially determined the jailed balloon diameter size according to the SB lumen size by visual estimation in CAG and positioned the proximal end of the jailed balloon at the ostium of SB. The purpose for SB dilatation is not mainly to dilate it to full expansion but to prevent SB compromise. For that purpose, we tended to choose smaller sized balloon catheter than the SB reference vessel diameter (RVD). Moreover, we always refer to the rule of Saito's paper that was determined to be half of the stent diameter in the MV. We decided on the length of the jailed balloon in accordance with the diseased lesion length of SB from its ostium. That is, the distal end of the jailed balloon was intended to be situated on the healthy site without any marked plaque. In most of our cases, the jailed balloon was 1.5 mm in diameter (80.0% in the HP group; 61.9% in the LP group), and the balloon length was 12 mm or smaller (93.3% in the HP group; 90.5% in the LP group). The size distribution of the jailed balloon for both the diameter and length was similar in each group. Thus, the third factor of “inflation pressure of the jailed balloon” became the subject of our interest in this study.

Here, we compared the procedural outcomes of the modified jailed balloon technique between the two groups in which the jailed balloon was inflated at a higher or lower pressure in SB. At first, we predicted that the higher inflation pressure might be more likely to cause arterial dissection in SB, and a lower inflation pressure might be more likely to fail to prevent carina shifting. However, no significant differences in procedural outcomes between the two groups were shown in our study. In particular, SB dissection and SB ostial stenosis increase occurred at similar frequencies irrespective of the inflation pressure of the jailed balloon in SB. one of the reasons may be that the dilation of a smaller sized jailed balloon (about 1.6 to 1.7-mm in diameter) with the pressure from average 5 to 10 atm did not result in a large diameter difference and led no major difference in SB compromise. In the conventional modified jailed balloon technique, to discriminate the inflation pressure between the jailed balloon in SB and the stent-mounted balloon in MV is reasonable to minimize the stent deformation due to balloon overlapping. However, because our modified jailed balloon technique has less interference between them, to discriminate the inflation pressure is considered to be less effective. On the other hand, as shown in previous reports, the native lesion characteristics must be risk factors of SB compromise [22]. In case 1, the mid-LAD lesion was very tortuous and the eccentric plaque was distributed on the opposite side of SB. This morphology is said to be associated with a high risk of causing carina shift when deploying a crossover stent in MV [23]. Volume reduction of the eccentric plaque using debulking devices such as rotational atherectomy (RA) or directional coronary atherectomy (DCA) can help reduce the risk of carina shift. Although we did not use any debulking devices in this case, we could maintain SB patency with the modified jailed balloon technique. However, some inevitable carina shift caused increasing stenosis at the SB ostium (Figure 1). In case 2, we encountered difficulty in guidewire advancement to SB after deploying the MV stent due to arterial dissection from the SB ostium. SB dissection was caused by jailed balloon inflation in the diseased SB. As mentioned above, the procedure of inflating a balloon in a diseased SB is associated with a risk of causing arterial dissection. In this situation, we have to take much care on performing guidewire recross into the SB to avoid enlarging the dissected lumen.

SB dissection easily occurred in the lesion with some plaque burden irrespective of the magnitude of the jailed balloon inflation pressure. The development of SB dissection was not necessarily localized at the SB ostium (Table 3). SB is relatively small in most cases, so the distal edge of the jailed balloon bears some risks of causing arterial dissection when it contacts the plaque. Our data suggest this fact that univariate analysis showed that SB lesion length (LL) ≥8.65 mm was significantly associated with SB compromise. Therefore, to prevent SB dissection as much as possible, it is more important to adopt a jailed balloon length that can cover the whole of the diseased lesion from the SB ostium. On the other hand, from this point of view, the noninflation modified jailed balloon technique may be one of the promising methods to minimize the risk of causing SB dissection though it may be less effective for preventing carina shift. We performed the noninflation jailed balloon procedure in three cases of the LP group. And no SB compromise was indeed observed in these cases. However, further examination with more cases is needed to determine the optimal method of jailed balloon inflation in the SB during the modified jailed balloon technique.

## 5. Limitation

This study has several limitations. The major one is that the design was a retrospective observational study of a small number of patients at a single center. We did not define the precise rule regarding case selection that needed a modified jailed balloon technique and the judgement was left to the operator's discretion, which might have affected the results of this study. Most aspects of the modified jailed balloon technique have already been established, but there are some procedural variations among facilities or interventional cardiologists. We performed this technique in accordance with the standard method at our hospital, as described in Materials and Methods, but we have not evaluated the superiority among those variations of this procedure, including our method. Moreover, we have not compared this technique with the jailed wire technique, one of the easiest and most conventional methods for protecting the SB opening. Previous reports showed that carina shift is a more important contributor to anatomical SB ostial compromise [20]. On the other hand, the anatomical significance is not closely associated with the functional significance in SB after MV stenting [24]. In our study, we focused on SB compromise, defined as described in Materials and Methods, after the modified jailed balloon technique in a provisional T stent strategy. Because this definition is weighted on anatomical evaluation, we might have overestimated the negative impact of SB compromise. Plaque modification with debulking devices such as RA or DCA has been demonstrated to be useful for preventing SB compromise, but we did not use them in any of our cases. The unavailability of those devices might have affected the frequency and severity of SB compromise.

## 6. Conclusions

The modified jailed balloon technique is one of the effective techniques to minimize the emergence of SB compromise by preventing plaque or carina shift during MV stent implantation. SB dissection developed in lesions with some plaque burden, irrespective of the magnitude of the jailed balloon inflation pressure. We speculate that lesion characteristics rather than the PCI procedural factors may be critical determinants to cause SB compromise.

## Figures and Tables

**Table 1:** Baseline clinical characteristics of the study patients.

	HP (*n* = 30)	LP (*n* = 21)	*P* value
Age (years old)	73.9 ± 12.2	72.0 ± 12.1	0.585
Male (%)	22 (73.3)	16 (76.2)	1
Hypertension (%)	21 (70.0)	16 (76.2)	0.754
Diabetes mellitus (%)	12 (40.0)	10 (47.6)	0.774
Dyslipidemia (%)	16 (53.3)	10 (47.6)	0.779
Smoking (%)	11 (36.7)	4 (19.0)	0.221
HD (%)	1 (3.3)	2 (9.5)	0.561
Af (%)	5 (16.7)	6 (28.6)	0.327
CVD (%)	5 (16.7)	6 (28.6)	0.327
PAD (%)	5 (16.7)	3 (14.3)	1
PCI indications			0.717
ACS (%)	9 (30.0)	4 (19.0)	
Stable AP (%)	9 (30.0)	7 (33.3)	
SMI (%)	12 (40.0)	10 (47.6)	

ACS: acute coronary syndrome, Af: atrial fibrillation, AP: angina pectoris, CVD: cerebral vascular disease, HD: hemodialysis, HP: higher pressure, LP: lower pressure, PAD: peripheral arterial disease, PCI: percutaneous coronary intervention, and SMI: silent myocardial ischemia.

**Table 2:** Angiographic lesion and procedural characteristics of the study patients.

	HP (*n* = 30)	LP (*n* = 21)	*P* value
Lesion location			0.756
Distal LMCA (%)	3 (10.0)	0 (0.0)	
LAD-D1 (%)	14 (46.7)	13 (61.9)	
LAD-D2 (%)	9 (30.0)	5 (23.8)	
LAD-D3 (%)	1 (3.3)	1 (4.8)	
LCX-OM, LCX-PL (%)	2 (6.7)	1 (4.8)	
RCA-RV (%)	1 (3.3)	1 (4.8)	
Medina classification			1
1, 1, 1 (%)	18 (60.0)	14 (66.7)	
1, 1, 0 (%)	1 (3.3)	1 (4.8)	
1, 0, 1 (%)	4 (13.3)	2 (9.5)	
0, 1, 1 (%)	6 (20.0)	4 (19.0)	
0, 0, 1 (%)	1 (3.3)	0 (0.0)	
Guide catheter			0.015
6Fr (%)	22 (73.3)	21 (100.0)	
7Fr (%)	8 (26.7)	0 (0.0)	
Imaging			1
IVUS (%)	22 (73.3)	16 (76.2)	
OCT/OFDI (%)	8 (26.7)	5 (23.8)	
MV lesion prep with CB (%)	16 (53.3)	10 (47.6)	0.779
MV predilation balloon diameter (%)	2.64 ± 0.31	2.62 ± 0.25	0.772
SB predilatation (%)	11 (36.7)	7 (33.3)	1
SB predilation balloon diameter (%)	1.89 ± 0.38	2.07 ± 0.19	0.105
Modified jailed balloon			
MV stent diameter (mm)	2.94 ± 0.36	2.83 ± 0.37	0.296
MV stent length (mm)	25.83 ± 7.39	25.71 ± 8.06	0.957
MV stent inflation pressure (atm)	10.8 ± 1.9	11.2 ± 1.4	0.371
SB jailed balloon diameter (mm)	1.64 ± 0.32	1.71 ± 0.30	0.416
SB jailed balloon length (mm)	12.27 ± 1.78	12.33 ± 1.20	0.882
SB jailed balloon inflation pressure (atm)	10.8 ± 1.9	5.4 ± 2.4	<0.05
Inflation frequency	2.5 ± 0.7	2.5 ± 0.5	0.957
Stent			0.142
SYNERGY (%)	12 (40.0)	3 (14.3)	
Xience Sierra (%)	9 (30.0)	7 (33.3)	
Ultimaster Tansei (%)	4 (13.3)	4 (19.0)	
Orsiro (%)	2 (6.7)	6 (28.6)	
Resolute onyx (%)	2 (6.7)	1 (4.8)	
BIOFREEDOM (%)	1 (3.3)	0 (0.0)	

CB: cutting balloon, D1: first diagonal branch, D2: second diagonal branch, D3: third diagonal branch, IVUS: intravascular ultrasound, LAD: left anterior descending, LCX: left circumflex, LMCA: left main coronary artery, MV: main vessel, OCT: optical coherence tomography, OFDI: optical frequency domain imaging, OM: obtuse marginal branch, PL: posterior-lateral branch, RCA: right coronary artery, RV: right ventricular branch, and SB: side branch.

**Table 3:** Procedural outcomes of modified jailed balloon technique.

	HP (*n* = 30)	LP (*n* = 21)	*P* value
Procedural success (%)	30 (100.0)	21 (100.0)	NS
SB compromise (%)	9 (30.0)	7 (33.3)	1
SB dissection			0.798
Ostium (%)	5 (16.7)	4 (19.0)	
Mid (%)	1 (3.3)	2 (9.5)	
Distal (%)	1 (3.3)	1 (4.8)	
SB ostial stenosis increase (%)	3 (10.0)	2 (9.5)	1
Temporary SB TIMI flow <grade 3 (%)	1 (3.3)	2 (9.5)	0.561
Jailed balloon or wire entrapment (%)	0 (0.0)	0 (0.0)	NS
GW recross (%)	29 (96.7)	20 (95.2)	1
SB dilatation (%)	24 (80.0)	13 (61.9)	0.207
SB provisional stenting	0 (0.0)	0 (0.0)	NS
KBT (%)	20 (66.7)	8 (38.1)	0.0522
POT (%)	10 (33.3)	2 (9.5)	0.445
Extra stent dilatation (%)	1 (5.0)	0 (0.0)	1
Peri procedural MI	0 (0.0)	0 (0.0)	NS
In hospital MACE (%)	0 (0.0)	0 (0.0)	NS

GW: guidewire, KBT: kissing balloon technique, MACE: major adverse cardiac event, MI: myocardial infarction, POT: proximal optimization technique, and TIMI: thrombolysis in myocardial infarction.

**Table 4:** QCA analysis of targeted lesions in the initial and final angiography.

Pre procedure	HP (*n* = 30)	LP (*n* = 21)	*P* value	Post procedure	HP (*n* = 30)	LP (*n* = 21)	*P* value
MV RVD (mm)	2.56 ± 0.42	2.44 ± 0.30	0.274	MV RVD (mm)	2.77 ± 0.40	2.59 ± 0.30	0.081
MV MLD (mm)	0.64 ± 0.46	0.65 ± 0.36	0.889	MV MLD (mm)	2.50 ± 0.40	2.30 ± 0.31	0.053
MV % DS (%)	75.49 ± 16.24	73.70 ± 12.97	0.678	MV % DS (%)	9.91 ± 4.45	11.33 ± 6.19	0.344
MV LL (mm)	18.59 ± 7.83	18.70 ± 6.98	0.961				
SB RVD (mm)	1.84 ± 0.38	1.78 ± 0.28	0.563	SB RVD (mm)	1.92 ± 0.41	1.86 ± 0.26	0.599
SB MLD (mm)	0.67 ± 0.42	0.64 ± 0.30	0.745	SB MLD (mm)	1.33 ± 0.55	1.21 ± 0.40	0.368
SB % DS (%)	64.16 ± 19.64	64.16 ± 16.07	1	SB % DS (%)	31.65 ± 20.23	34.84 ± 21.28	0.591
SB LL (mm)	8.42 ± 4.01	6.67 ± 3.16	0.100				

DS: diameter stenosis, LL: lesion length, MLD: minimal lumen diameter, QCA: quantitative coronary angiography, and RVD: reference vessel diameter.

**Table 5:** Univariate and multivariate analysis for SB compromise.

	Univariate		Multivariate	
	OR [95% CI]	*P* value	OR [95% CI]	*P* value
HP group	1.16 [0.29–4.50]	1		
SB lesion preparation (+)	1.68 [0.41–6.72]	0.529		
MV calcification (+)	9.65 [1.21–449.44]	0.019	8.74 [0.99–76.90]	0.051
SB RVD ≥ 1.79 mm	0.29 [0.06–1.20]	0.071		
SB MLD ≤ 0.54 mm	2.76 [0.71–11.66]	0.131		
SB LL ≥ 8.65 mm	4.20 [1.03–18.48]	0.027	3.77 [0.99–14.40]	0.052

CI: confidence interval, DS: diameter stenosis, HP: higher pressure, LL: lesion length, MV: main vessel, OR: odds ratio, RVD: reference vessel diameter, and SB: side branch.

## Data Availability

The data used to support the findings of this study are available from the corresponding author upon request.

## Supplementary Materials

Supplementary Materials Original raw data of 51 patients of this study and the results of statistical analysis based on the patients' raw data are provided.
