# Supplementary material for: Inflation Pressure in Side Branch during Modified Jailed Balloon Technique Does Not Affect Side Branch Outcomes
Source: J Interv Cardiol. 2021 Feb 18;2021:8839897. doi: 10.1155/2021/8839897 (PMC7906823; doi:10.1155/2021/8839897)

	A	B	Fisher検定 p値
F	8	5	1
M	22	16	

ACS	9	4	0.724
AP	9	7	
OMI	3	4	
SMI	9	6	

ACS	9	4	0.717
AP	9	7	
SMI	12	10	

HT (-)	9	5	0.754
HT(+)	21	16	

DM(-)	18	11	0.774
DM(+)	12	10	

DL(-)	14	11	0.779
DL(+)	16	10	

smoke(-)	19	17	0.221
smoke(+)	11	4	

HD(-)	29	19	0.561
HD(+)	1	2	

Af (-)	25	15	0.327
Af (+)	5	6	

CVD(-)	25	15	0.327
CVD(+)	5	6	

PAD(-)	25	18	1
PAD(+)	5	3	

	A	B	Fisher検定 p値
CCB(-)	15	7	0.266
CCB(+)	15	14	

ACEi(-)	17	12	1
ACEi(+)	13	9	

BB(-)	26	13	0.0515
BB(+)	4	8	

Loop(-)	26	15	0.283
Loop(+)	4	6	

Spiron(-)	28	20	1
Spiron(+)	2	1	

Statin (-)	19	10	0.389
Statin (+)	11	11	

Insulin (-)	29	18	0.293
Insulin (+)	1	3	

ASA(-)	0	1	0.412
ASA(+)	30	20	

Plasg(-)	5	9	0.0572
Plasg(+)	25	12	

Clopid(-)	25	12	0.0572
Clopid(+)	5	9	

DOAC(-)	28	17	0.214
DOAC(+)	2	4	

WBC	平均	標準偏差	p値	F検定 p値
A	7152.333	2615.301	0.968	0.0222
B	7114.286	4162.486		
Hgb	13.74	1.725189	0.0417	0.0888
	12.52381	2.433907		
Plt	227.6	63.74118	0.688	0.77
	220.0952	67.35793		
Egfr	69.46	26.0865	0.123	0.211
	56.35714	33.55367		
Alb	3.986667	0.5781858	0.0751	0.923
	3.685714	0.5867587		
BS	144.0667	66.96625	0.923	0.343
	146.0952	80.98945		
HDL	50.06	19.30357	0.236	0.0195
	44.39524	11.52937		
LDL	108.83	33.78936	0.366	0.538
	99.32381	29.53892		
BNP	239.3567	474.0407	0.135	0.00372
	523.9952	857.4611		
CRP	0.55	1.150337	0.184	0.00000174
	1.390952	3.141579		
年齢	73.9	12.20613	0.585	0.973
	72	12.05819		

	A	B	Fisher検定 p値
LAD-D1	14	13	0.756
LAD-D2	9	5	
LAD-D3	1	1	
LCX	2	1	
LMT	3	0	
RCA-RV	1	1	

LAD	24	19	0.613
LCX	2	1	
LMT	3	0	
RCA	1	1	

1,1,1	18	14	1
1,1,0	1	1	
1,0,1	4	2	
0,1,1	6	4	
0,0,1	1	0	

MV calc			
+	22	14	0.757
−	8	7	

IVUS	22	16	1
OCT/OFDI	8	5	

6Fr	22	21	0.015
7Fr	8	0	

CB

+	16	10	0.779
−	14	11	

SC

+	3	1	0.634
−	27	20	

Lesion prep (SB)

+	11	7	1
−	19	14	

Stent	A	B	0.142
BIOFREED	1	0	
Orsiro	2	6	
Resolute O	2	1	
SYNERGY	12	3	
Ultimaster	4	4	
Xience	9	7	

Diameter (MV)	平均	標準偏差	p値
A	2.642857	0.307533	0.772
B	2.619048	0.245192	

Diameter (SB)

A	1.886364	0.376889	0.249
B	2.071429	0.188982	

JBT

Diameter (stent)

A	2.941667	0.357695	0.296
B	2.833333	0.365148	

length (stent)

A	25.83333	7.386303	0.957
B	25.71429	8.05694	

pressure (MV)

A	10.8	1.864366	0.371
B	11.2381	1.445848	

Diameter (JB)

A	1.641667	0.319505	0.416
B	1.714286	0.298807	

length (JB)

A	12.26667	1.779836	0.882
B	12.33333	1.197219	

pressure (SB)

A	10.8	1.864366	5.23E-12
B	5.428571	2.378475	

Inf. Time

A	2.533333	0.681445	0.957
B	2.52381	0.511766	

	F検定 p値
	0.3
	0.105
	0.901
	0.656
	0.24
	0.767
	0.0691
	0.226
	0.186

GW recross

+	29	20	1
−	1	1	

SB dilatation

+	24	13	0.207
−	6	8	

Final KBT

+	20	8	0.0522
−	10	13	

KBT/POT

+	21	10	0.148
−	9	11	

KBT

+	20	8	0.0522
−	10	13	

POT

+	6	2	0.445
−	24	19	

	A	B	Fisher検定 p値
SB compromise (+)	9	7	1
SB compromise (−)	21	14	
dissection (+)	7	7	0.529
dissection (−)	23	14	
stenosis increase (+)	3	2	1
stenosis increase (−)	27	19	
dissection (-)	23	14	0.798
dissection (os)	5	4	
dissection (body)	1	2	
dissection (distal edge	1	1	
Temporary SB TIMI flow <grade 3			0.561
(-)	1	2	
(+)	29	19	

MV ref (pre)	平均	標準偏差	p値	F検定 p値
A	2.562	0.419683	0.274	0.136
B	2.443333	0.303765		
MV MLD (pre)				
A	0.6363333	0.464962	0.889	0.241
B	0.6533333	0.360643		
MV %DS (pre)				
A	75.48639	16.23888	0.678	0.299
B	73.70452	12.97093		
MV LL (pre)				
A	18.594	7.827307	0.961	0.6
B	18.6981	6.977182		
SB ref (pre)				
A	1.839	0.379058	0.563	0.159
B	1.782381	0.279229		
SB MLD (pre)				
A	0.673	0.41987	0.745	0.116
B	0.6380952	0.298507		
SB %DS (pre)				
A	64.16427	19.63714	1	0.354
B	64.16383	16.0677		
SB LL (pre)				
A	8.422	4.010861	0.1	0.27
B	6.665714	3.158303		

MV ref (post)	平均	標準偏差	p値	F検定 p値
A	2.774333	0.400015	0.081	0.201
B	2.59	0.303183		
MV MLD (post)				
A	2.501	0.395643	0.053	0.273
B	2.295714	0.312083		
MV %DS (post)				
A	9.912187	4.445931	0.344	0.102
B	11.33275	6.185932		
SB ref (post)				
A	1.915	0.414211	0.599	0.0305
B	1.860952	0.257525		
SB MLD (post)				
A	1.334667	0.552597	0.368	0.141
B	1.20619	0.401404		
SB %DS (post)				
A	31.65472	20.23411	0.591	0.788
B	34.83819	21.28266		

	Univariate			Multivariate		
	OR	95%CI	P	OR	95%CI	P
Category	1.163121	0.2930602 - 4.5039994	1			
Lesion prep	1.6789	0.4147585 - 6.7204887	0.5294			
MV calc	9.648699	1.209669 - 449.438347	0.01937	8.74	0.994 - 76.90	0.0506
SB RVD≧1.79mm	0.2877913	0.05625479 - 1.19508762	0.07128			
SB MLD≦0.54mm	2.761174	0.7121058- 11.6569239	0.1314			
LL≧8.65mm	4.198205	1.029026 - 18.479538	0.0271	3.77	0.99 - 14.4	0.0518

	オッズ比	95%信頼区間下限	95%信頼区間上限	P値
(Intercept)	0.0475	0.00561	0.402	0.00519
MV.calc[T.+]	8.7400	0.99400	76.900	0.05060
LL.8.65[T.+]	3.7700	0.99000	14.400	0.05180

SB RVD　曲線下面積 0.719　95%信頼区間 0.568 - 0.87

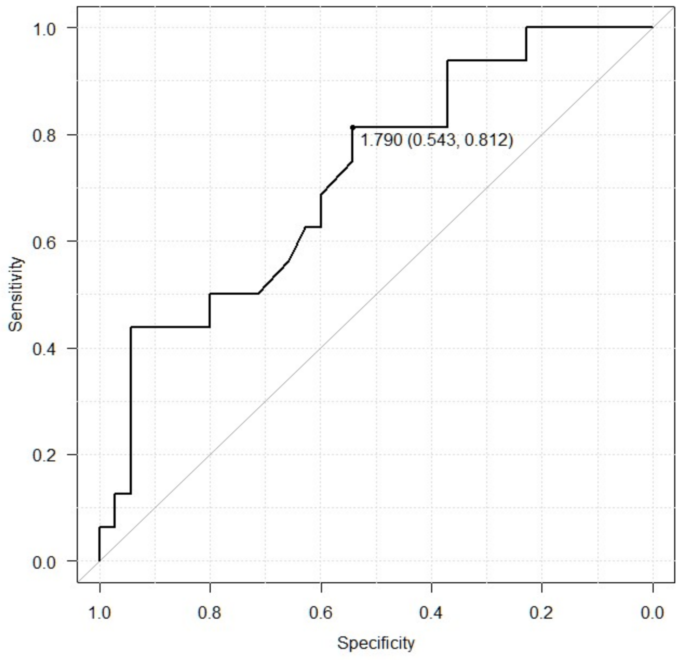

SB MLD　曲線下面積 0.664　95%信頼区間 0.514 - 0.814

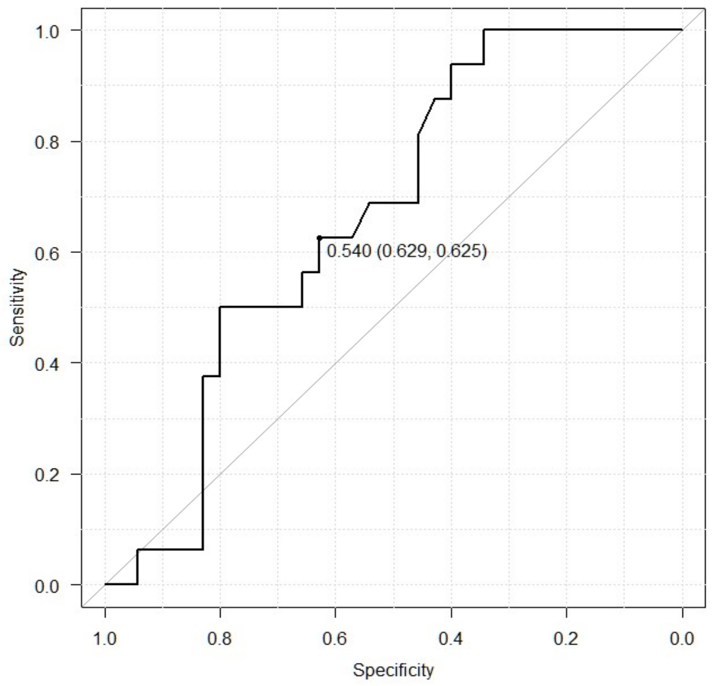

SB LL　曲線下面積 0.678　95%信頼区間 0.521 - 0.834

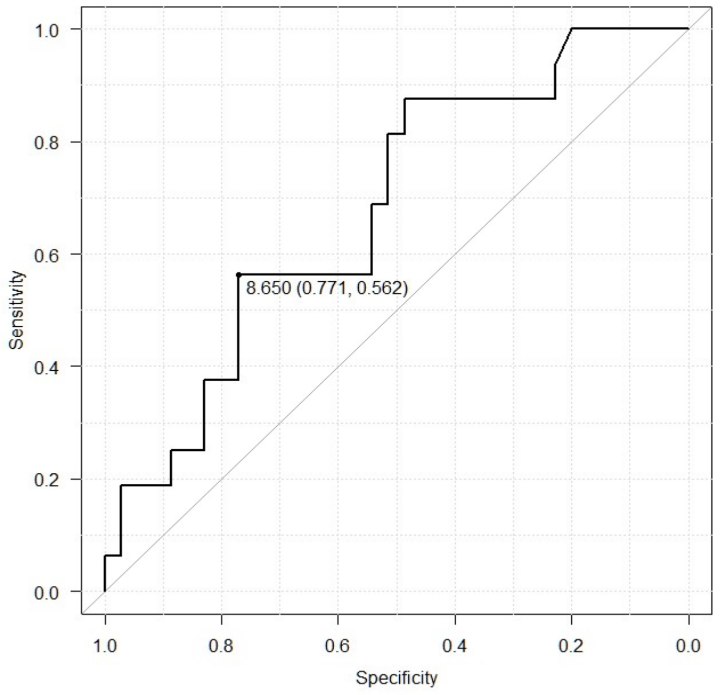

Supplement: Supplementary Materials — Original raw data of 51 patients of this study and the results of statistical analysis based on the patients' raw data are provided. [file 8839897.f1.zip › 8839897.f1/Analysis data.pdf]
