# Supplementary material for: Inflation Pressure in Side Branch during Modified Jailed Balloon Technique Does Not Affect Side Branch Outcomes
Source: J Interv Cardiol. 2021 Feb 18;2021:8839897. doi: 10.1155/2021/8839897 (PMC7906823; doi:10.1155/2021/8839897)
Supplement: Supplementary Materials — Original raw data of 51 patients of this study and the results of statistical analysis based on the patients' raw data are provided. [file 8839897.f1.zip › 8839897.f1/raw data.pdf]

No.	Date	Age	Sex	ACS	Lesion	Medina	MV calc	GC size	Image	Lesion prep (MV)	Lesion prep (SB)	pre KBT	MV stent	Jailed balloon	Pressure (MV/SB)	Inf. Time	Recross GW	Ext. St. Dil.	SB balloon	MV balloon
1	2018/11/26	84	M		LAD-D2	1,1,1	+	6Fr	Alta	Wolverine 2.5/10	–	–	SYNERGY 2.75/20	Regnam 1.5/12	12atm/12atm	2	Sion	–	SC 1.5/15	NC 2.75/15
2	2018/12/23	71	M	ACS	LAD-LCX	1,1,1	+	7Fr	Opti	NC 3.0/15	–	–	Xience 3.5/15	Ryurei 2.0/20	12atm/12atm	1	Sion	–	SC 2.0/20	NC 3.5/15
3	2018/12/28	81	M		LAD-D1	1,0,1	+	6Fr	OCT	SC 2.5/15	–	–	SYNERGY 3.0/20	Regnam 1.5/12	12atm/12atm	3	Fielder FC	–	SC 2.5/12	NC 3.25/15
4	2019/1/1	77	M	ACS	LAD-D1	0,1,1	+	6Fr	Alta	Wolverine 3.0/10	–	–	Ultimaster3.0/24	Regnam 1.5/12	12atm/12atm	2	Fielder FC	–	SC 2.0/20	NC 3.5/15
5	2019/1/7	50	M	ACS	LAD-D2	1,1,1	+	6Fr	Alta	SC 2.75/15	–	–	SYNERGY 3.0/28	Regnam 1.5/12	10atm/10atm	2	Fielder FC	–	SC 2.0/15	NC 3.0/15
6	2019/1/29	93	M	ACS	LAD-D2	1,1,1	+	7Fr	Alta	SC 2.5/15	SC 2.0/20	+	SYNERGY 3.0/24	Regnam 1.5/12	12atm/12atm	3	Sion black	–	SC 2.0/20	NC 3.25/15
7	2019/1/30	42	M	ACS	LAD-HL	1,1,1	–	7Fr	Opti	SC 3.0/15	SC 2.0/15	+	SYNERGY 4.0/32	Regnam 2.0/15	10atm/10atm	1	Fielder FC	–	SC 3.0/15	NC 3.75/15
8	2019/2/1	70	M		LAD-D1	1,1,1	+	6Fr	Alta	Wolverine 2.75/10	SC 1.5/15	–	Xience 3.0/38	Regnam 1.5/12	12atm/12atm	1	Sion black	–	SC 1.5/12	NC 3.5/15
9	2019/4/8	80	M		LAD-D1	0.1.1	–	6Fr	OCT	SC 2.5/15	–	–	Xience 3.0/33	Regnam 1.5/12	12atm/8atm	2	Fielder FC	–	SC 1.5/12	NC 3.0/16
10	2019/6/28	89	F		RCA-RV	1,1,1	–	6Fr	OCT	SC 2.0/15	–	–	Orsiro 2.25/26	Regnam 1.5/12	12atm/6atm	2	Fielder FC	–	SC 2.5/6	NC 2.5/16
11	2019/7/23	50	M		LAD-D2	0.1.1	–	6Fr	OCT	Wolverine 2.25/10	–	–	Orsiro 2.5/22	Regnam 1.5/12	12atm/6atm	2	Fielder FC	–	–	–
12	2019/8/5	99	M		LAD-D1	0,1,1	+	6Fr	Opti	Wolverine 2.5/10	–	–	SYNERGY 3.0/38	Regnam 1.5/12	11atm/11atm	3	Fielder FC	–	SC 1.5/12	NC 3.0/16
13	2019/8/5	75	F		LAD-D2	1,1,1	+	6Fr	Alta	NSE 2.5/13	SC 1.5/10	–	BIOFREEDOM 3.0/28	Sapphire 1.5/10	6atm/6atm	3	XT-R	–	balloon not crossed	NC 3.0/15
14	2019/8/15	84	M		LAD-D2	1,1,1	+	6Fr	Alta	NC 2.75/10	SC 2.5/10	–	SYNERGY 2.5/24	Ikazuchi 2.5/10	11atm/6atm	3	Fielder FC	–	SC 2.5/10	NC 2.75/10
15	2019/8/22	70	M	ACS	LAD-D1	1,1,1	+	6Fr	Alta	SC 2.5/15	–	–	SYNERGY 3.0/28	Regnam 1.5/12	12atm/6atm	2	Fielder FC	–	SC 2.5/6	NC 3.25/16
16	2019/9/5	62	M		LAD-D2	1,1,1	+	6Fr	Alta	Wolverine 2.75/10	–	–	SYNERGY 3.0/38	Regnam 1.5/12	11atm/11atm	3	Fielder FC	–	SC 1.5/12	NC 3.0/15
17	2019/9/13	63	M		LAD-D1	1,1,0	+	6Fr	Opti	SC 2.75/15	–	–	Orsiro 3.0/30	Regnam 1.5/12	12atm/6atm	2	Fielder FC	–	–	NC 3.5/15
18	2019/10/10	84	M		LCX-PL	1,1,1	–	6Fr	Navifocus	SC 2.0/10	SC 2.0/10	–	R-Onyx 2.5/26	Regnam 1.5/12	12atm/12atm	2	Fielder FC	–	SC 1.5/12	SC 2.5/15
19	2019/10/11	59	F		LCX-OM	1,0,1	+	6Fr	Opti	Wolverine 2.75/10	–	–	Xience 2.75/15	Regnam 1.5/12	12atm/6atm	2	Fielder FC	–	SC 1.5/15	NC 2.5/10
20	2019/10/23	73	M		LAD-D1	1,0,1	+	6Fr	Alta	Wolverine 3.0/10	SC 2.0/15	–	Orsiro 3.0/40	Regnam 2.0/15	12atm/8atm	2	Fielder FC	–	–	NC 3.0/15
21	2019/10/24	62	M		LAD-D1	1,0,1	+	6Fr	Opti	SC 2.5/15	–	–	Orsiro 3.0/30	Regnam 2.0/12	8atm/8atm	2	Wizard78	+	SC 2.0/20→Wolverine 2.0/10	NC 3.0/15
22	2019/11/1	59	M		LAD-D2	1,1,1	–	6Fr	Alta	SC 2.75/15	–	–	Xience 3.0/38	Regnam 1.5/12	11atm/6atm	3	Fielder FC	–	SC 1.5/12	NC 3.0/15
23	2019/11/22	71	M	ACS	LAD-D2	1,1,1	+	6Fr	Alta	SC 2.5/15	SC 2.0/15	–	UM Tansei 3.0/33	Regnam 2.0/15	9atm/6atm	2	Fielder FC	–	SC 2.0/15	NC 3.0/15
24	2019/11/28	72	M	ACS	LAD-D1	1,1,1	–	6Fr	Alta	Wolverine 3.0/10	–	–	Xience 4.0/28	Regnam 2.0/12	12atm/6atm	2	Sion black	–	SC 2.0/12	NC 4.0/15
25	2019/12/9	73	F		LAD-D1	1,0,1	+	6Fr	OCT	Wolverine 2.5/10	SC 1.5/12	–	Xience Sierra 2.5/18	Regnam 1.5/12	6atm/6atm	3	Fielder FC	–	SC 1.5/12	NC 2.75/15
26	2019/12/11	84	F		LAD-D1	1,0,1	+	6Fr	Alta	Wolverine 2.5/10	SC 1.5/12	–	SYNERGY 2.5/38	Regnam 1.5/12	12atm/12atm	3	Fielder FC	–	SC 1.5/12	NC 2.5/15
27	2020/1/7	90	F		LAD-D1	1,1,1	+	6Fr	Alta	Wolverine 2.5/10	–	–	Xience Sierra 2.5/33	Regnam 2.0/12	9atm/6atm	3	Fielder FC	–	SC 2.0/12	NC 2.75/15
28	2020/1/14	79	F		LAD-D2	1,1,1	+	6Fr	EagleEye	Wolverine 2.5/10	SC 2.0/15	–	Orsiro 2.5/18	Ignis 2.0/15	8atm/0atm	2	Fielder FC	–	SC 2.0/15	NC 2.5/15
29	2020/1/24	72	M		LM bif.	0,1,1	+	7Fr	OCT	NSE 3.0/13	NC 2.75/15	–	SYNERGY 3.0/20	Regnam 2.75/12	11atm/11atm	3	Fielder FC	–	SC 2.75/12	NC 3.25/15
30	2020/1/29	81	M		LAD-D1	1,1,1	+	6Fr	Alta	Wolverine 3.0/10	SC 2.0/12	–	SYNERGY 3.0/12	Regnam 2.0/12	11atm/6atm	3	Fielder FC	–	SC 2.0/12	NC 3.0/12
31	2020/1/31	61	M		LAD-D1	0,1,1	–	6Fr	OCT	NSE 2.75/13	–	–	UM Tansei 2.75/18	Regnam 1.5/12	10atm/6atm	3	Fielder FC	–	SC 1.5/12	NC 3.0/15
32	2020/2/6	77	M		LAD-D1	1,1,1	+	6Fr	OCT	NC 2.5/16	–	–	Orsiro 3.0/18	Regnam 1.5/12	12atm/6atm	3	FC not cross	–	–	–
33	2020/2/25	71	M		LAD-D1	0,1,1	+	6Fr	Alta	Wolverine 2.75/10	NSE 2.0/13	–	UM Tansei 3.0/33	Regnam 1.5/12	12atm/8atm	3	Fielder FC	–	–	NC 3.25/16
34	2020/3/10	90	F		LAD-D1	0,1,1	+	6Fr	Opti	Wolverine 2.5/10	–	–	UM Tansei 2.5/33	Regnam 1.5/12	10atm/10atm	2	Fielder FC	–	SC 1.5/12	NC 3.5/15
35	2020/3/11	72	F		LAD-D1	1,1,1	+	6Fr	Alta	Wolverine 2.5/10	–	–	R-Onyx 2.5/22	Regnam 1.5/12	14atm/6atm	3	Fielder FC	–	SC 1.5/12	NC 2.5/15
36	2020/3/19	86	M		LAD-D2	0,1,1	–	6Fr	Alta	SC 2.5/30	SC 2.0/20	–	SYNERGY 3.0/24	Regnam 1.5/12	11atm/11atm	3	Fielder FC	–	balloon not crossed	NC 3.0/15
37	2020/4/3	84	M		LAD-D1	1,1,1	+	6Fr	Alta	Wolverine 2.75/10	–	–	Xience Sierra 3.0/33	Regnam 1.5/12	12atm/0atm	3	Fielder FC	–	–	NC 3.0/15
38	2020/4/9	72	M	ACS	LAD-D3	1,1,1	–	7Fr	Alta	SC 2.0/15	SC 2.0/15	–	Orsiro 2.5/30	Regnam 1.5/12	8atm/8atm	2	Fielder FC	–	SC 1.5/12	NC 2.25/15
39	2020/4/23	79	M		LCX-OM	0,1,1	+	6Fr	OCT	Wolverine 2.5/10	–	–	Xience Sierra 2.75/15	Regnam 1.5/12	12atm/12atm	3	Fielder FC	–	SC 1.75/10	NC 2.75/15
40	2020/4/28	46	M	ACS	LAD-D3	1,1,1	–	6Fr	Alta	Wolverine 2.5/10	–	–	UM Tansei 2.5/21	Regnam 1.5/12	9atm/0atm	3	Fielder FC	–	–	NC 3.0/15
No.	Date	Age	Sex		Lesion	Medina	MV calc	GC size	Image	Lesion prep (MV)	Lesion prep (SB)	pre KBT	MV stent	Jailed balloon	Pressure (MV/SB)	Inf. Time	Recross GW	Ext. St. Dil.	SB balloon	MV balloon
41	2020/6/3	64	M		LAD-D2	1,1,1	+	6Fr	OCT	NSE 2.75/13	SC 2.0/15	–	Xience Sierra 3.0/18	ZINRAI 1.5/10	12atm/12atm	3	Sion black	–	–	NC 3.25/15
42	2020/6/25	81	M		LAD-D1	1,1,1	+	6Fr	Alta	SC 2.5/15	SC 2.0/12	+	Xience Sierra 2.75/15	Regnam 2.0/12	12atm/6atm	3	Fielder FC	–	–	NC 2.75/12
43	2020/9/1	76	M		LAD-D1	1,1,0	+	6Fr	OFDI	Wolverine 2.5/10	–	–	SYNERGY 2.5/24	Regnam 2.5/15	11atm/11atm	3	Gaia next1	–	SC 2.5/15	NC 2.75/12
44	2020/9/4	71	F		LAD-D1	1,1,1	–	6Fr	OCT	Wolverine 2.5/10	–	–	UM Tansei 2.75/12	Regnam 1.5/12	9atm/9atm	3	black not cross	–	–	NC 2.75/10
45	2020/9/8	71	M		LAD-D2	1,1,1	–	6Fr	NIRS	Wolverine 2.5/10	–	–	Xience Sierra 3.0/28	Regnam 1.5/12	12atm/12atm	3	Fielder FC	–	–	NC 3.0/15
46	2020/9/21	74	F	ACS	LAD-D1	0,0,1	–	7Fr	Alta	–	–	–	R-Onyx 3.5/18	Regnam 1.5/12	12atm/12atm	3	Fielder FC	–	SC 1.5/12	NC 3.5/15
47	2020/10/2	85	F		LAD-D1	1,1,1	+	6Fr	OCT	Wolverine 2.5/10	–	–	Xience Sierra 2.75/18	ZINRAI 1.5/10	14atm/14atm	2	Fielder FC	–	SC 1.5/10	NC 2.75/16
48	2020/10/21	80	M		LAD-D2	1,1,1	+	6Fr	Alta	Wolverine 2.75/10	–	–	SYNERGY 3.0/32	Regnam 1.5/12	11atm/11atm	3	Fielder FC	–	–	NC 3.25/16
49	2020/11/14	65	M	ACS	RCA-RV	1,1,1	–	7Fr	Alta	–	–	–	Xience Sierra 3.5/30	Regnam 1.5/12	12atm/12atm	3	Fielder FC	–	SC 1.5/15	NC 3.5/15
50	2020/12/11	64	M	ACS	LAD-D1	1,1,1	+	7Fr	Alta	SC 2.75/15	–	–	Xience Skypoint 3.25/28	Regnam 2.0/12	12atm/12atm	3	Sion black	–	SC 2.0/12	NC 3.5/15
51	2020/12/18	61	F		LAD-D1	1,1,1	+	6Fr	Alta	SC 3.0/15	–	–	UM Tansei 2.5/28	Regnam 1.5/12	9atm/9atm	3	Fielder FC	–	SC 2.25/15	NC 2.75/15
No.	Date	Age	Sex		Lesion	Medina	MV calc	GC size	Image	Lesion prep (MV)	Lesion prep (SB)	pre KBT	MV stent	Jailed balloon	Pressure (MV/SB)	Inf. Time	Recross GW	Ext. St. Dil.	SB balloon	MV balloon

		SB Outcomes						QCA pre procedure								QCA post procedure			
KBT	POT	SB compromise	dissection	dis	stenosis-increase	SB flow disturbance	SB final TIMI	MV ref	MV MLD	MV %DS	LL	SB ref	SB MLD	SB %DS	LL	MV ref	MV MLD	MV %DS	SB ref
+	–		–	–	–	3	3	2.28	0.64	71.92982	12.63	1.65	0.31	81.21212	11.75	2.9	2.6	10.34483	1.67
+	NC 4.0/15		–	–	–	3	3	3.39	0.89	73.74631	12.14	2.11	0.99	53.08057	16.6	3.36	3.2	4.761905	3.06
+	–		–	–	–	3	3	2.56	0.73	71.48438	18.85	1.58	0.75	52.53165	11.29	2.98	2.77	7.04698	1.6
–	–	dis grade 2 (ostium)	+	dis (os)	–	3	3	2.77	0.57	79.42238	20.06	1.51	0.34	77.48344	6.43	3.07	2.76	10.09772	1.53
+	–		–	–	–	3	3	2.02	0.45	77.72277	15.87	1.56	0.58	62.82051	7.36	2.15	2.02	6.046512	1.45
+	–		–	–	–	3	3	2.75	0.01	99.63636	16	1.36	0.01	99.26471	8.47	2.85	2.71	4.912281	1.55
+	NC 5.0/8		–	–	–	3	3	3.35	0.71	78.80597	16.92	2.36	1.31	44.49153	10.03	3.65	3.3	9.589041	2.45
–	–	dis grade 2 (distal edge)	+	dis (dis)	–	3	3	2.93	0.36	87.71331	29.53	1.91	0.41	78.53403	19.59	3.11	2.41	22.50804	1.9
+	–		–	–	–	3	3	2.72	0.71	73.89706	24.97	1.76	0.4	77.27273	9.49	2.93	2.83	3.412969	1.88
–	–		–	–	–	3	3	2.19	0.36	83.56164	15.55	1.67	0.49	70.65868	3.21	2.24	2.01	10.26786	1.69
–	NC 2.75/8		–	–	–	3	3	2.37	0.61	74.2616	16.1	1.63	0.56	65.64417	8.35	2.54	2.34	7.874016	1.73
+	–	stenosis increase	–	–	+	3	3	1.98	0.51	74.24242	25	1.79	0.73	59.21788	3.99	2.38	2.03	14.70588	1.79
–	–	stenosis increase	–	–	+	3	2	2.27	0.62	72.68722	23.67	1.39	0.34	75.53957	9.2	2.29	1.98	13.53712	1.38
+	–	dis grade 2 (body)	+	dis (body)	–	3	3	1.91	0.23	87.95812	21.19	1.54	0.39	74.67532	9.72	2.21	1.74	21.26697	1.93
+	–	stenosis increase	+	dis (os)	+	3	3	2.55	0.52	79.60784	14.59	1.59	0.54	66.03774	15.35	2.86	2.39	16.43357	1.65
+	–		–	–	–	3	3	2.53	0.87	65.61265	30.83	2.05	1.18	42.43902	6.74	2.75	2.67	2.909091	2.06
–	–		–	–	–	3	3	2.91	1.52	47.76632	26.27	1.8	1.5	16.66667	1.88	2.69	2.42	10.03717	1.94
+	–		–	–	–	3	3	2.12	0.22	89.62264	22.71	2.62	0.43	83.58779	3.01	2.54	2.24	11.81102	2.3
–	–	dis grade 2 (ostium)	+	dis (os)	–	(+)	3	2.19	0.7	68.03653	9.14	1.32	0.5	62.12121	7.33	2.23	2.16	3.139013	1.26
–	–		–	–	–	3	3	2.74	1.37	50	33.2	1.87	0.49	73.79679	4.41	2.77	2.14	22.74368	1.96
–	–	dis grade 2 (ostium)	+	dis (os)	–	(+)	3	2.6	1.27	51.15385	23.27	1.49	0.41	72.48322	8.88	2.97	2.66	10.43771	1.93
+	–		–	–	–	3	3	2.48	0.68	72.58065	20.29	2.32	0.76	67.24138	2.58	2.57	2.02	21.40078	2.14
–	–		–	–	–	3	3	2.46	0.89	63.82114	24.87	2.06	0.62	69.90291	8.45	2.64	2.04	22.72727	2.14
+	–		–	–	–	3	3	3.03	0.41	86.46865	19.87	1.76	1.1	37.5	5.78	3.28	3.06	6.707317	1.94
+	–	dis grade 2 (ostium)	+	dis (os)	–	3	3	2.02	0.51	74.75248	15.53	1.41	0.38	73.04965	8.65	2.56	2.16	15.625	1.5
+	–		–	–	–	3	3	2.22	0.19	91.44144	28.83	1.63	0.62	61.96319	5.54	2.25	2.03	9.777778	1.57
+	–	dis grade 2 (ostium) + stenosis increase	+	dis (os)	+	3	3	2.32	0.38	83.62069	30.86	1.73	0.83	52.02312	4.32	2.76	2.48	10.14493	2.26
–	–		–	–	–	3	3	2.33	0.82	64.80687	13.2	1.87	0.21	88.77005	8.07	2.34	2.28	2.564103	1.98
+	NC 4.5/6		–	–	–	3	3	3.21	0.9	71.96262	10.51	3.05	1.7	44.2623	8.17	3.53	3.29	6.798867	3.04
–	–	dis grade 2 (distal edge)	+	dis (dis)	–	(+)	3	2.82	0.67	76.24113	10.91	2.01	0.78	61.19403	10	2.97	2.65	10.77441	1.91
+	–		–	–	–	3	3	2.47	1.28	48.17814	13.82	2.31	0.82	64.50216	5.09	2.5	2.3	8	2.17
–	NC 3.25/8		–	–	–	3	3	2	0.69	65.5	12.51	1.83	0.96	47.54098	4.93	2.04	1.87	8.333333	1.9
–	–		–	–	–	3	3	2.74	0.54	80.29197	27	1.4	0.24	82.85714	7.83	2.8	2.48	11.42857	1.44
–	–		–	–	–	3	3	2.29	0.36	84.27948	30.66	1.55	1.16	25.16129	2.47	2.83	2.49	12.01413	1.65
+	–	dis grade 2 (body)	+	dis (body)	–	3	3	2.12	0.35	83.49057	17.46	1.52	0.58	61.84211	7.81	2.5	2.25	10	1.56
–	–		–	–	–	3	3	2.69	0.33	87.73234	20.71	1.62	0.55	66.04938	8.2	2.78	2.49	10.43165	1.66
–	–		–	–	–	3	3	2.14	0.28	86.91589	19.08	1.55	0.43	72.25806	3.99	2.71	2.39	11.80812	1.55
+	–		–	–	–	3	3	2.07	0.25	87.92271	23.23	1.69	0.31	81.6568	5.33	2.26	2.13	5.752212	1.68
+	–		–	–	–	3	3	2.06	0.43	79.12621	4.76	2.04	1.34	34.31373	2.58	2.24	1.91	14.73214	2.14
–	–		–	–	–	3	3	2.28	0.28	87.7193	10.64	2.24	0.48	78.57143	3.81	2.25	2	11.11111	2.02
KBT	POT	SB compromise	dissection	dis	stenosis-increase	SB flow disturbance	SB final TIMI	MV ref	MV MLD	MV %DS	LL	SB ref	SB MLD	SB %DS	LL	MV ref	MV MLD	MV %DS	SB ref
–	–	dis grade 2 (body)	+	dis (body)	–	3	3	2.91	0.54	81.4433	13.26	2.09	0.1	95.21531	11.16	2.9	2.69	7.241379	2.32
–	–	dis grade 2 (ostium)	+	dis (os)	–	3	3	2.54	0.43	83.07087	11.14	1.65	0.72	56.36364	7.58	2.56	2.36	7.8125	2.03
+	NC 3.5/10	stenosis increase	–	–	+	3	3	2.48	1.02	58.87097	8.52	1.58	1.09	31.01266	5.43	2.52	2.18	13.49206	1.71
–	–	dis grade 2 (ostium)	+	dis (os)	–	3	3	2.45	0.74	69.79592	5.98	1.67	0.34	79.64072	7.14	2.49	2.18	12.4498	1.76
–	–		–	–	–	3	3	2.66	0.35	86.84211	19.94	1.84	0	100	10.68	2.77	2.66	3.971119	1.79
+	–		–	–	–	3	3	2.9	2.44	15.86207	2.63	2.07	0.53	74.39614	4.42	3.45	3.2	7.246377	2.2
+	–		–	–	–	3	3	2.56	1.17	54.29688	11.35	1.88	1.08	42.55319	5.06	2.75	2.39	13.09091	1.86
–	–	dis grade 2 (ostium)	+	dis (os)	–	3	3	2.71	1.05	61.25461	28.37	1.76	0.75	57.38636	14.54	2.65	2.37	10.56604	1.76
+	–		–	–	–	3	3	3.22	0.61	81.0559	24.32	2.08	0.98	52.88462	7.88	3.04	2.57	15.46053	1.94
+	–		–	–	–	3	3	2.89	0.12	95.84775	24.48	2.18	0.8	63.30275	12.75	2.91	2.72	6.52921	2.22
+	NC 4.0/15		–	–	–	3	3	1.97	0.23	88.32487	17.26	1.65	0.67	59.39394	9.32	2.3	2.22	3.478261	1.98
KBT	POT	SB compromise	dissection	dis	stenosis-increase	SB flow disturbance	SB final TIMI	MV ref	MV MLD	MV %DS	LL	SB ref	SB MLD	SB %DS	LL	MV ref	MV MLD	MV %DS	SB ref

SB MLD	SB %DS
1.04	37.72455
2.22	27.45098
1.25	21.875
1.17	23.52941
0.7	51.72414
1.42	8.387097
2.3	6.122449
0.83	56.31579
1.14	39.3617
1.32	21.89349
1.48	14.45087
0.54	69.8324
0.27	80.43478
1.8	6.735751
0.3	81.81818
1.84	10.67961
1.8	7.216495
1.79	22.17391
1.18	6.349206
1.19	39.28571
1.69	12.43523
1.56	27.1028
1.09	49.06542
1.54	20.61856
0.84	44
0.92	41.40127
0.97	57.07965
0.68	65.65657
2.72	10.52632
1.59	16.75393
1.1	49.30876
1.13	40.52632
0.61	57.63889
1.36	17.57576
1.1	29.48718
0.75	54.81928
0.89	42.58065
1.25	25.59524
1.92	10.28037
1.81	10.39604
SB MLD	SB %DS
0.94	59.48276
1.05	48.27586
1.05	38.59649
0.96	45.45455
1.3	27.3743
1.05	52.27273
1.76	5.376344
1.2	31.81818
1.67	13.91753
1.58	28.82883
1.71	13.63636
SB MLD	SB %DS
